# Hepatic changes during a carrageenan induced granuloma in rats

**DOI:** 10.1155/S0962935193000110

**Published:** 1993

**Authors:** J. Muntané, Y. Fernandez, S. Mitjavila, M. T. Mitjavila

**Affiliations:** 1 Unitat de Fisiologia Departament de Bioquímica i Fisiologia Facultat de Biologia Av. Diagonal 645 Barcelona 08028 Spain; 2 Institut de Physiologie 2 rue F. Magendie Toulouse 31400 France

## Abstract

Hepatic changes during inflammation were studied in rats bearing a carrageenan induced granuloma. In spite of a decrease in the metabolic capacity of microsomes to induce lipid peroxidation during inflammation, the endogenous lipid peroxidation remained unchanged and unrelated with the hepatic activities measured. The continuous increase in hepatic cAMP observed during acute and chronic phases could be related to adenylate cyclase stimulation by mediators, and could be an initial step in the hepatocyte adaptation leading to the increased level of hepatic caeruloplasmin, to the reduction of cytochrome P-450 level and to the modifications of Ca^2+^ sequestration by microsomes.


Mediators of Inflammation, 2, 79–83 (1993)

### Hepatic changes during a carrageenan induced granuloma in rats

J. Muntané,[1] Y. Fernandez,[2] S. Mitjavila[2] and M. T. Mitjavila[1,CA]

[1]Unitat de Fisiologia, Departament de Bioquímica i Fisiologia, Facultat de Biologia, Av. Diagonal 645, 08028-Barcelona, Spain. [2]Institut de Physiologie, 2 rue F. Magendie, 31400-Toulouse, France

[CA] Corresponding Author

**HEPATIC changes during inflammation were studied in rats bearing a carrageenan induced granuloma. In spite of a decrease in the metabolic capacity of microsomes to induce lipid peroxidation during inflammation, the endogenous lipid peroxidation remained unchanged and unrelated with the hepatic activities measured. The continuous increase in hepatic cAMP observed during acute and chronic phases could be related to adenylate cyclase stimulation by mediators, and could be an initial step in the hepatocyte adaptation leading to the increased level of hepatic caeruloplasmin, to the reduction of cytochrome P-450 level and to the modifications of $Ca^{2+}$ sequestration by microsomes.**

**Key words:** $Ca^{2+}$ sequestration, Caeruloplasmin, cAMP, Cytochrome P-450, Inflammation, Lipid peroxidation, Liver, Microsomes

### Introduction

It is becoming evident that the liver has an important role in the development of inflammatory processes.[1–3] Physiological adaptive changes have been observed, such as a decrease in hepatic smooth reticuloendothelial system drug metabolism during adjuvant induced arthritis[4–6] and during carrageenan induced granulomatous inflammation in rats.[7] A reduction in cytochrome P-450 dependent activities has been observed after administration of IL-6.[8] The cytochrome P-450 system seems to have a central role in the control of hepatic activities during inflammation. It is also known that lipid peroxidation reduces the level of cytochrome P-450[9,10] and microsomal $Ca^{2+}$ sequestration,[9] but the role of peroxidative degradation of liver phospholipids during inflammation remains poorly understood and controversial.[7,11,12] In previous papers, the authors reported that carrageenan induced granuloma increased the thiobarbituric (TBA) reactive substances in plasma and in the inflammatory exudate.[13,14] Mediators and reactants released locally can also modify the physiological activity of hepatocytes via the stimulation of adenylate cyclase.[23]

The purpose of this research was to study the relation between inflammation and the regulation of several hepatic microsomal activities. Thus, particular attention has been paid to the changes in endogenous lipid peroxidation and in cAMP in relation to the synthesis of acute phase proteins such as caeruloplasmin, to $Ca^{2+}$ sequestration, and to cytochrome P-450 levels.

### Materials and Methods

*Animals and materials:* Three groups of nine male Sprague–Dawley rats, each weighing approximately 225 g were used. The animals were maintained in accordance with the NIH Guide for the Care and Use of Laboratory Animals (NIH Publications No. 80-23, 1978) in the Animal Care Centre of the Faculty of Biology. One group was used as control (Control) and the other two were killed at 1 (I-1 day) and 6 days (I-6 days) after the induction of a carrageenan granuloma, enabling discrimination between the acute and chronic phases of the inflammation. The granuloma was induced by a s.c. injection of 6 ml of air into the dorsum of the rats, followed 24 h later by 4 ml of carrageenan 2% (w/v) (Viscarin 402, Marine Colloids, Springfield, NJ, USA) in NaCl 0.9%.[15] The three groups were starved overnight before the experiment, but given water *ad libitum*.

Blood was obtained by cardiac puncture under ether anaesthesia with a heparinized syringe and plasma was separated after centrifugation at 1 800 × *g* at 4°C for 10 min.

The liver was perfused with NaCl 0.9% through the subhepatic vein and weighed, and 0.1 and 2.0 g were immediately removed for cAMP

© 1993 Rapid Communications of Oxford Ltd assay and for isolation of the microsomal fraction respectively.

NADP, ATP, isocitrate and isocitrate dehydrogenase were supplied by Sigma Chemical Co (St Louis, MO). $^{45}Ca^{2+}$ was obtained as aqueous $^{45}CaCl_2$ from the I.R.E. (Fleurus, Belgium). All other chemicals and reagents were of the highest purity available.

*Caeruloplasmin assay:* The caeruloplasmin level was evaluated in 0.1 ml of plasma by measurement of its *p*-phenylenediamine oxidase activity, according to the Sunderman and Nomoto technique.[16]

*Hepatic cAMP assay:* A 0.1 g sample of liver was homogenized in 10 ml of methanol/acetic acid (95:5). After centrifugation at 2 000 × ***g*** for 10 min the protein concentration in 50 $\mu$l of supernatant was measured by the method of Lowry *et al.*[17] using bovine serum albumin as a standard. The remaining supernatant was stored at −80°C until cAMP assay was carried out by radioimmunoassay (RIA, $^{125}$I-cAMP, Immunotech, France) in a fraction containing 250 $\mu$g of protein.

*Hepatic microsomal assays:* The hepatic microsomal fraction from 2 g of liver sample was obtained by the method of Lowrey *et al.*,[18] and the protein concentration was measured.[16] Cytochrome P-450 was evaluated by the capacity of its reduced form to react with CO.[19] $Ca^{2+}$ sequestration was measured as described by Moore.[20] Endogenous lipid peroxidation was determined by the absorbance at 233 nm (conjugated dienes) of the extracted lipids dissolved in cyclohexane.[21] The metabolic capacity of microsomes to induce peroxidation was assayed after 30 min of incubation at 37°C in the presence of an NADPH generating system,[18] $CCl_4$ or $FeSO_4$, at the concentrations given in Table 2, and the TBA reactive substances produced were measured by the method of Ghoshal and Recknagel.[22]

### Results

The concentration of caeruloplasmin in plasma (Fig. 1) increased 1 day after the induction of the granuloma (0.60 g/l), and even more after 6 days (1.13 g/l) compared to the control value (0.34 g/l). Figure 2 shows a significant increase in the relative weight of the liver 1 day after the induction of the inflammation. No difference was found between the values obtained at 1 day and at 6 days of inflammation. cAMP concentration in the liver (Fig. 3) increased gradually throughout the development of the inflammation, reaching 1.25 pg/mg protein 6 days after the induction.

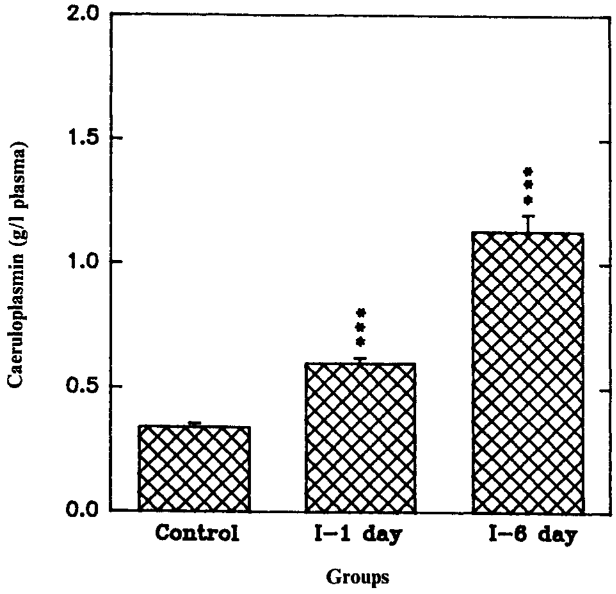

FIG. 1. Changes in the plasma concentration of caeruloplasmin induced by inflammation. Groups and assay were as described in Materials and Methods. Data represent mean ± S.E.M. of nine rats. The results were analyzed using Student's *t* test. Inflamed groups (I) were compared with control group (Control): *$p < 0.05$, **$p < 0.01$, ***$p < 0.001$.

At the hepatic microsomal membrane level, no significant difference between groups was found in the endogenous conjugated diene concentration (Table 1). The level of cytochrome P-450 (Table 1) decreased strongly after the induction of the inflammation (55% at I-1 day and 60% at I-6 day). $Ca^{2+}$ sequestration by the microsomal fraction (Table 1) was substantially affected by the

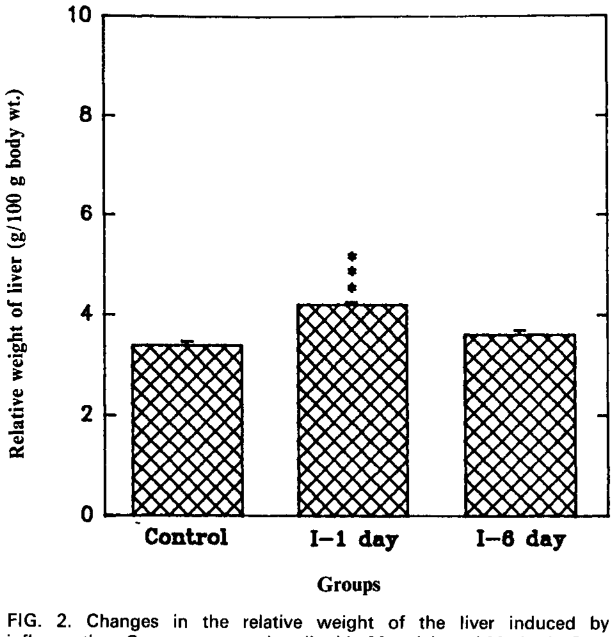

FIG. 2. Changes in the relative weight of the liver induced by inflammation. Groups were as described in Materials and Methods. Data represent mean ± S.E.M. of nine rats. The results were analyzed using Student's *t* test. Inflamed groups (I) were compared with control group (Control): *$p < 0.05$, **$p < 0.01$, ***$p < 0.001$.

**Table 1** Effect of inflammation on the level of conjugated dienes, cytochrome P-450 and $Ca^{2+}$ sequestration in the hepatic microsomal fraction

| Groups | Conjugated dienes (absorbance at 233 nm of 1 mg lipids/ml cyclohexane) | Cytochrome P-450 (nmol/mg prot.) | $Ca^{2+}$ sequestration (nmol/mg prot. per 30 min) |
|---|---|---|---|
| Control | 0.288 ± 0.0092 | 0.963 ± 0.0265 | 85 ± 4.8 |
| I-1 day | 0.311 ± 0.0158 | 0.433 ± 0.0222*** | 146 ± 4.1*** |
| I-6 day | 0.313 ± 0.0141 | 0.382 ± 0.0577*** | 58 ± 9.1*** |

Data represent mean ± S.E.M. of nine rats. The results were analyzed using Student's *t*-test. Inflamed groups (I) were compared with control group (Control). $^{*}p < 0.05$, $^{**}p < 0.01$, $^{***}p < 0.001$.

inflammation, reaching the highest value 1 day after the induction (146 nmol $Ca^{2+}$/mg protein) and showing a lower value 6 days after induction (58 nmol $Ca^{2+}$/mg protein) than the control group (85 nmol $Ca^{2+}$/mg protein).

The metabolic capacity of microsomes to induce lipid peroxidation decreased during inflammation (Table 2) when incubated in the presence of 1.5 $\mu$M $FeSO_4$ or 0.5 $\mu$M $CCl_4$. The metabolic activity of microsomes in the presence of both inducers was lower in I-6 day than in I-1 day groups.

### Discussion

The increase in the relative weight of the liver during the acute phase of inflammation suggests intense activity of the Kupffer cells and it had been observed that administration of zymosan intravenously[23] and rhamnose intraperitoneally[24] stimulated the reticuloendothelial system.

Some parameters show a change under the effect of inflammation while others, such as conjugated dienes, remain unchanged. In other situations it has been postulated that lipid peroxidation is responsible for a reduction in the activity of cytochrome P-450.[9,25] As to its role in inflammation, Robak[11] observed a reduction in the TBA reactive substances in the homogenate of the liver during inflammation induced by carrageenan (3 and 24 h after induction) and by Freund's adjuvant (1, 5, 10 and 21 days after induction). However, other authors reported an increase in the TBA reactive substances in the homogenate of the liver, at 21 days after adjuvant induced arthritis[12] and 7 days after carrageenan induced granuloma growing around subcutaneously implanted teflon chambers.[7] In our conditions the relation between microsomal lipid peroxidation and the decrease of cytochrome P-450 is difficult to assume since there is no difference in conjugated diene concentration between groups, although the authors have observed an increase of TBA reactive substances in plasma and in the inflammatory exudate.[13,14] So, there may

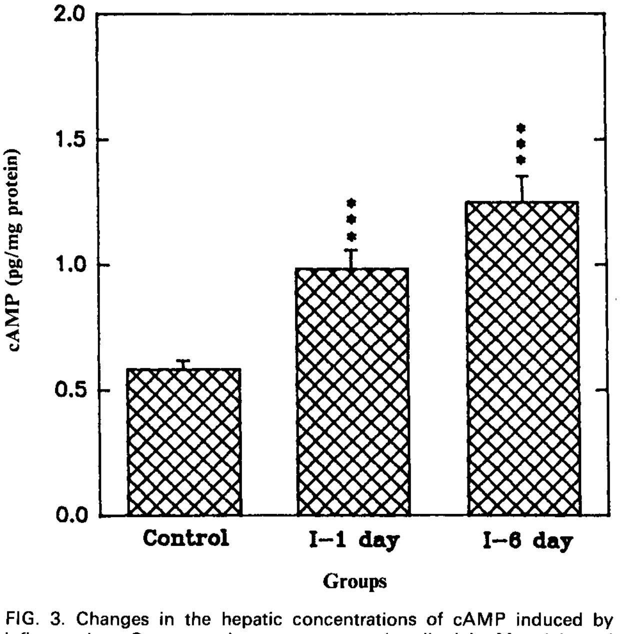

FIG. 3. Changes in the hepatic concentrations of cAMP induced by inflammation. Groups and assay were as described in Materials and Methods. Data represent mean ± S.E.M. of nine rats. The results were analyzed using Student's *t* test. Inflamed groups (I) were compared with control group (Control): $^{*}p < 0.05$, $^{**}p < 0.01$, $^{***}p < 0.001$.

**Table 2.** Effect of $FeSO_4$ and $CCl_4$ on the level of TBA-reactive substances generated by hepatic microsomal fraction

| Groups | TBA reactive substances (nmol malondialdehyde/mg protein) | |
|---|---|---|
| | $FeSO_4$(1.5 $\mu$M) | $CCl_4$(0.5 $\mu$M) |
| Control | 5.56 ± 0.314 | 1.330 ± 0.0149 |
| I-1 day | 3.59 ± 0.309** | 0.505 ± 0.0546*** |
| I-6 day | 2.89 ± 0.211*** | 0.097 ± 0493*** |

Data represent mean ± S.E.M. of nine rats. The results were analyzed using Student's *t* test. Inflamed groups (I) were compared with control group (Control). $^{*}p < 0.05$, $^{**}p < 0.01$, $^{***}p < 0.001$.

be other mechanisms for the down-regulation of cytochrome P-450. Fukuda *et al.*[24] postulated haem deficiency which results from the induction of haem oxygenase or the increase in specific cytokines such as IL-6, but the phosphorylation of cytochrome P-450 by adrenalin and glucagon may also be involved.[27]

In the present work an increase in hepatic cAMP while cytochrome P-450 decreased. Inflammatory mediators and hepatocytes may thus be involved in the increase in cAMP. On the other hand, adrenalin and glucagon (factors present during acute phase reactions) added to hepatocytes in culture increased the phosphorylation of certain isoenzymes of cytochrome P-450.[7,28,29] It has thus been postulated that the increase in hepatic cAMP may be responsible for the phosphorylation of cytochrome P-450 and its degradation,[29] transforming the P-450 form to the inactive form P-420.[30] Such a mechanism could lead to changes in cytochrome P-450 concentration during inflammation and also to the decrease in its capacity to induce lipid peroxidation in the presence of $FeSO_4$ or $CCl_4$, which is in accordance with studies carried out by Sakai *et al.*[31] showing that IL-1 treatment depresses superoxide production by microsomes. This study will be pursued in order to clarify the role of local and systemic mediators (Il-1, TNF, catecholamines, glucocorticoids) *in vitro*.

The increase in the $Ca^{2+}$ sequestration by microsomes during the acute phase of inflammation suggests an important role for this element as second intracellular messenger. It has been observed that the capacity of microsomes to sequestrate cytosolic $Ca^{2+}$ is modified by glucagon or cAMP analogues.[32,33]

Caeruloplasmin, as an acute phase protein, is a good indicator of inflammatory response.[34] The transcription of the caeruloplasmin gene in the liver is stimulated by several factors (catecholamines, IL-6, glucocorticoids) where cAMP is the intracellular messenger involved.[3] So, the increase in hepatic cAMP during inflammation may cause high concentrations of caeruloplasmin in plasma.

These results suggest that mediators released during carrageenan induced inflammation could increase cAMP in hepatocytes and then stimulate synthesis of caeruloplasmin, reduce the level of cytochrome P-450, and modify $Ca^{2+}$ sequestration by the microsomal fraction.

### References

1. Decker K. Hepatic mediators of inflammation. In: Wisse E, Knook DL, Decker K, eds. *Cells of the Hepatic Sinusoids.* The Netherlands: Kupffer Cell Foundation, 1989; 171–175.
2. Dinarello CA. Interleukin-1. *Rev Infect Dis* 1984; **6:** 51–95.
3. Cousins RJ. Absorption, transport, and hepatic metabolism of copper and zinc: special reference to mallothionein and ceruloplasmin. *Physiol Rev* 1985; **65:** 238–309.
4. Beck FJ, Whitehouse MW. Effect of adjuvant disease in rats on cyclophosphamide and isophosphamide metabolism. *Biochem Pharmacol* 1973; **22:** 2453–2468.
5. Cawthorne MA, Palmer ED, Green J. Adjuvant-induced arthritis and drug-metabolizing enzymes. *Biochem Pharmacol* 1976; **25:** 2683–2688.
6. Morton DM, Chatfield DH. The effect of adjuvant induced arthritis on the liver metabolism of drugs in rats. *Biochem Pharmacol* 1970; **19:** 473–481.
7. Adolfs MJP, Bonta IL, Bragt PC. Increased lipid peroxidation and decreased hepatic aminopyrine metabolism during carrageenin-induced granulomatous inflammation in the rat. *Proceedings of the Biochemical Pharmacology Symposium* 1979; 12–14 September, 123P–124P.
8. Chen YI, Florentin I, Batt AM, Ferrari L, Giroud JP, Chauvelot-Moachon L. Effects of interleukin-6 on cytochrome P450-dependent mixed function oxidases in the rat. *Biochem Pharmacol* 1992; **44:** 137–148.
9. Cambon-Gros C, Carrera G, Mitjavila S. Inhibition of $Ca^{2+}$ sequestration in foetal liver microsomes by carbon tetrachloride and bromotrichloromethane. *Biochem Pharmacol* 1984; **33:** 2605–2608.
10. Mori T, Kitada M, Imaoka S, Funae Y, Kamataki T. The *in vitro* effects of lipid peroxidation on the content of individual forms of cytochrome P-450 in liver microsomes of guinea-pigs. *Pharmacol Res* 1991; **2:** 143–148.
11. Robak J. Adjuvant-induced and carrageenin-induced inflammation and lipid peroxidation in rat liver, spleen and lungs. *Biochem Pharmacol* 1978; **27:** 531–533.
12. Ishizuki S, Kujihira E. Peroxidative status of isolated hepatocytes from adjuvant arthritic rats. *Res Commun Chem Pathol Pharmacol* 1984; **44:** 431–443.
13. Carbonell T, Sáiz MP, Mitjavila MT, *et al.* Carrageenan-induced granuloma and iron status in rats with dietary polyunsaturated fatty acid deficiency. *Br J Nutr* 1991; **65:** 497–503.
14. Puig-Parellada P, Carbonell MT, Sáiz MP, *et al.* Modulatory role of iron and polyunsaturated fatty acids in inflammation. *Pharmacol (Life Sci Adv)* 1990; **9:** 509–518.
15. Fukuhara M, Tsurufuji S. The effect of locally injected anti-inflammatory drugs on the carrageenin granuloma in rats. *Biochem Pharmacol* 1969; **18:** 475–484.
16. Sunderman FW, Nomoto S. Measurement of human serum ceruloplasmin by its *p*-phenylenediamine oxidase activity. *Clin Chem* 1970; **16:** 903–910.
17. Lowry OH, Rosebrough NJ, Farr AL, Randall RJ. Protein measurement with the Folin phenol reagent. *J Biol Chem* 1951; **193:** 265–275.
18. Lowrey K, Glende EA, Recknagel RO. Destruction of liver microsomal calcium pump activity by carbon tetrachloride and bromotrichloromethane. *Biochem Pharmacol* 1981; **30:** 135–140.
19. Omura T, Sato R. The carbon monoxide-binding pigment of liver microsomes. *J Biol Chem* 1964; **239:** 2370–2378.
20. Moore L. Inhibition of liver-microsome calcium pump by *in vivo* administration of $CCl_4$, $CHCl_3$ and 1,1-dichloroethylene. *Biochem Pharmacol* 1980; **29:** 2502–2511.
21. Recknagel, RO, Glende EA. Spectrophotometric detection of lipid conjugated dienes. In: Packer L. ed. *Oxygen radicals in Biological Systems.* Methods in Enzymology. Orlando: Academic Press, 1984; 331–337.
22. Ghoshal AK, Recknagel RO. Positive evidence of acceleration of lipoperoxidation in rat liver by carbon tetrachloride: *in vitro* experiments. *Life Sci* 1965; **4:** 1521–1530.
23. Sieck R, Roth B, Vahs W. Kinetics of rat RES by intravenously injected zymosan. In: Wisse E, Knook DL. eds. *Kupffer Cells and other Liver Sinusoidal Cells.* Amsterdam: Elsevier/North-Holland Biomedical Press, 1977; 407–415.
24. Wahl SM. Hepatic granuloma as a model of inflammation and repair: an overview. In: Di Sabato G. ed. *Immunochemical Techniques: Part M, Chemotaxis and Inflammation. Methods in Enzymology.* San Diego: Academic Press, 1988; 605–622.
25. Kitada M, Komori M, Ohi H. Form-specific degradation of cytochrome P-450 by lipid peroxidation in rat liver microsomes. *Res Commun Chem Pathol Pharmacol* 1989; **63:** 175–188.
26. Fukuda Y, Ishida N, Noguchi T, Kappas A, Sassa S. Interleukin-6 down regulates the expression of transcripts encoding cytochrome P450 *IA1, IA2* and *IIIA3* in human hepatoma cells. *Biochem Biophys Res Commun* 1992; **184:** 960–965.
27. Johansson I, Eliasson E, Ingelman-Sundberg M. Hormone controlled phosphorylation and degradation on CYT2B1 and CYPE1 in isolated hepatocytes. *Biochem Biophys Res Commun* 1991; **174:** 37–42.
28. Mkrtchian SL, Andersson KK. A possible role of cAMP dependent phosphorylation of hepatic microsomal cytochrome P450: A mechanism to increase lipid peroxidation in response to hormone. *Biochem Biophys Res Commun* 1990; **166:** 787–793.
29. Eliasson E, Johansson I, Ingelman-Sundberg M. Substrate-, hormone- and cAMP-regulated cytochrome P-450 degradation. *Proc Natl Acad Sci USA* 1990; **87:** 3225–3229.
30. Taniguchi H, Pyerin W, Stier A. Conversion of hepatic microsomal cytochrome P-450 to P-420 upon phosphorylation by cyclic AMP dependent protein kinase. *Biochem Pharmacol* 1985; **34:** 1835–1837.
31. Sakai H, Okamoto T, Yamamoto R, Sindhu RK, Kikkawa Y. Suppressive effect of interleukin-1 on pulmonary cytochrome P450 and superoxide anion production. *Biochem Biophys Res Commun* 1992; **185:** 1083–1090.
32. Andia-Waltenbaught AM, Lam A, Hummel L, Friedmann N. Characterization of the hormone-sensitive $Ca^{2+}$ uptake activity of the hepatic endoplasmic reticulum. *Biochim Biophys Acta* 1980; **630:** 165–175.

33. Mauger JP, Claret M. Mobilization of intracellular calcium by glucagon and cyclic AMP analogues in isolated rat hepatocytes. *FEBS Lett* 1986; **195**: 106–110.
34. Gutteridge JMC. Antioxidant properties of the proteins caeruloplasmin, albumin and transferrin. A study of their activity in serum and synovial fluid from patients with rheumatoid arthritis. *Biochim Biophys Acta* 1986; **869**: 119–127.

ACKNOWLEDGEMENTS. The authors wish to thank DGICYT (PM 89-0054) and Integrated Action between Spain and France (40/1989) for their financial support. The English version has been corrected by Robin Rycroft of the Language Advisory Service of the University of Barcelona.

**Received 10 November 1992; accepted in revised form 8 December 1993**
